# Identification of prognostic hub genes and functional role of BAIAP2L2 in prostate cancer progression: a transcriptomic and experimental study

**DOI:** 10.3389/fimmu.2025.1543476

**Published:** 2025-04-28

**Authors:** Xiangyang Zhan, Wenkai Wang, Jie Lian, Yichun Li, Jianyi Gu, Dongdong Guo, Dongliang Xu, Guanqun Ju

**Affiliations:** ^1^ Urology Center, Shuguang Hospital Affiliated to Shanghai University of Traditional Chinese Medicine, Shanghai, China; ^2^ Surgical Institute of Integrative Medicine, Shuguang Hospital Affiliated to Shanghai University of Traditional Chinese Medicine, Shanghai, China; ^3^ Surgical Institute, Shuguang Hospital Affiliated to Shanghai University of Traditional Chinese Medicine, Shanghai, China; ^4^ Shanghai Key Laboratory of Traditional Chinese Clinical Medicine, Shanghai, China; ^5^ Department of Oncology, Shuguang Hospital Affiliated to Shanghai University of Traditional Chinese Medicine, Shanghai, China; ^6^ Department of Plastic and Reconstructive Surgery, Shanghai Ninth People‘s Hospital, Shanghai JiaoTong University School of Medicine, Shanghai, China; ^7^ School of Basic Medical Sciences, Xinxiang Medical University, Xinxiang, Henan, China

**Keywords:** prostate cancer, BAIAP2L2, transcriptomic analysis, gene expression, biomarkers, therapeutic targets

## Abstract

**Background:**

Prostate cancer (PCa) is a prevalent malignancy in men, and understanding its molecular mechanisms is crucial for identifying therapeutic targets.

**Methods:**

Transcriptomic data from prostate tumors and matched healthy tissues were obtained from The Cancer Genome Atlas (TCGA). Differential expression analysis using the DESeq2 algorithm identified differentially expressed genes (DEGs). Cox proportional hazards regression was used to evaluate prognostic significance. Clinical validation involved comparing tumor specimens with normal tissues, focusing on BAIAP2L2, which showed significant differential expression and was further examined via immunohistochemical analysis. *In vitro* knockdown experiments were conducted in PC3 and DU145 cell lines to assess BAIAP2L2’s functional role through assays for migration, colony formation, and proliferation.

**Results:**

A total of 1,449 DEGs were identified, including 775 upregulated and 674 downregulated genes. Prognostic analysis revealed 748 genes linked to clinical outcomes, with 19 hub genes identified. QPCR confirmed significant upregulation of four candidates, including BAIAP2L2, which was also elevated in malignant tissues. BAIAP2L2 knockdown significantly impaired migration, proliferation, and viability in PCa cells.

**Conclusion:**

This study highlights crucial molecular mechanisms in PCa progression, particularly the significance of BAIAP2L2 as a potential therapeutic target, warranting further investigation into additional hub genes for effective targeted strategies.

## Introduction

Prostate cancer (PCa) is one of the most prevalent malignancies affecting men worldwide, and it represents a significant health burden due to its high incidence and potential for mortality (1, 2). According to the World Health Organization (WHO), PCa is the second most common cancer in men and the fifth leading cause of cancer-related deaths globally (3). In 2020 alone, there were over 1.4 million new cases and approximately 375,000 deaths attributed to PCa (4). PCa is often asymptomatic in its early stages, leading to a significant proportion of patients being diagnosed at advanced stages, frequently with bone metastases, which severely compromises patient survival (5). Although androgen deprivation therapy (ADT) is initially effective, the majority of patients eventually progress to castration-resistant prostate cancer (CRPC) (6). Alternative treatment modalities such as endocrine therapy, radiotherapy, surgery, and targeted drugs are available, they often result in substantial side effects without significantly improving patient prognosis (7). Therefore, there is an urgent need to develop novel diagnostic biomarkers and therapeutic targets for PCa.

The etiology of PCa is multifactorial, involving a complex interplay of genetic, environmental, and lifestyle factors. Age is one of the most significant risk factors; the likelihood of developing PCa increases substantially after the age of 50, with the majority of cases diagnosed in men over 65 (8). Family history and genetics also play a crucial role, as men with a first-degree relative (father, brother) diagnosed with PCa are at higher risk (9). Specific genetic mutations, such as those in the BRCA1 and BRCA2 genes, as well as variations in the HOXB13 gene, have been linked to an increased susceptibility to the disease (10). Ethnicity is another important determinant, with incidence rates varying significantly among different populations. African American men have the highest incidence and mortality rates of PCa compared to men of other ethnic backgrounds, whereas Asian and Hispanic men tend to have lower rates (11). The reasons for these disparities are not fully understood but are believed to involve a combination of genetic predispositions and socio-economic factors, including access to healthcare and differences in diet and lifestyle. Lifestyle factors, such as diet and physical activity, have been extensively studied for their role in PCa risk. Diets high in red and processed meats, high-fat dairy products, and low in fruits and vegetables have been associated with an increased risk of developing PCa (12). Conversely, a diet rich in fruits, vegetables, and healthy fats, such as those found in fish and nuts, may offer a protective effect (13). Obesity and metabolic syndrome are also significant risk factors, with numerous studies linking higher body mass index (BMI) and waist circumference to an elevated risk of aggressive PCa (14). Hormonal imbalances, particularly involving androgens (male hormones), are known to influence PCa development. Androgens, such as testosterone, stimulate prostate cell growth, and elevated levels have been associated with a higher risk of PCa (15). Additionally, conditions such as chronic inflammation and infections of the prostate (prostatitis) may contribute to carcinogenesis by causing DNA damage and promoting a pro-tumorigenic environment (16). The interplay of these risk factors underscores the complexity of PCa etiology and highlights the need for a multifaceted approach to prevention and early detection. Advances in understanding the molecular mechanisms underlying PCa have paved the way for new diagnostic markers and therapeutic targets, aiming to improve patient outcomes and reduce the burden of this disease.

In this investigation, we employed a comprehensive, multi-faceted approach to elucidate critical biomarkers in PCa. Transcriptomic data obtained from TCGA were systematically analyzed to identify differentially expressed genes (DEGs) between PCa and normal tissues using the DESeq2 algorithm, followed by prognostic evaluation through Cox proportional hazards regression analysis. Clinical validation was subsequently performed using patient specimens, leading to the identification of BAIAP2L2 as a pivotal biomarker. Functional characterization of BAIAP2L2 was conducted through *in vitro* experiments, wherein specific shRNA-mediated knockdown in PC3 and DU145 PCa cell lines enabled assessment of its impact on cellular migration, proliferation, and survival rates. This integrated methodological approach facilitated the delineation of BAIAP2L2’s role in PCa pathogenesis and explored its potential as a therapeutic target.

## Methods

### Data source

The transcriptomic data for prostate tumor and healthy tissues were downloaded and curated from The Cancer Genome Atlas (TCGA) database (https://portal.gdc.cancer.gov). Specifically, RNA sequencing data from the TCGA-PRAD (Prostate Adenocarcinoma) project processed using the STAR pipeline were extracted in TPM (Transcripts Per Million) format. Subsequently, these data were transformed using log2(value+1). The GWAS data for PCa were sourced from the TOPMed study, including 5,993 cases and a total sample size of 174,992 European individuals. Analyses for PCa GWAS were conducted using SAIGE (Scalable and Accurate Implementation of Generalized mixed model), a generalized mixed model association test that employs the saddlepoint approximation to account for case-control imbalance (17). Adjustments were made for genetic relatedness, sex, birth year, and the first four principal components.

### Transcriptomic data analysis

The transcriptomic data for prostate tumor and healthy tissues were analyzed using the DESeq2 R package. DEGs were identified with a threshold of log2 fold change (log2FC) ≥ 2 and adjusted p-value (P.adj) ≤ 0.05. A volcano plot was created to visualize the DEGs. Subsequently, clinical data corresponding to these samples were extracted, and samples lacking clinical information were excluded. The survival R package was used to perform proportional hazards assumption tests and to fit the Cox proportional hazards regression model. Genes with a p-value (P) ≤ 0.05 were considered to have prognostic value. The union of DEGs and prognostically significant genes was extracted for summary-data-based mendelian randomization. The DEGs will undergo KEGG (Kyoto Encyclopedia of Genes and Genomes) and GO (Gene Ontology) pathway analysis. KEGG is a comprehensive database that integrates genomic, chemical, and systemic functional information (18). It facilitates the understanding of high-level functions and utilities of the biological system, such as cells, organisms, and ecosystems, from molecular-level information. GO provides a framework for the model of biology by defining classes used to describe gene function, and relationships between these concepts, across all species (19). It encompasses three domains: Biological Process, Cellular Component, and Molecular Function. Then we utilized the GSEA software (http://www.broadinstitute.org/gsea/index.jsp) to perform Gene Set Enrichment Analysis (GSEA) on the MSigDB (c2.all.v7.5.1.symbols.gmt [Curated/Pathway]) gene sets.

The intersection of DEGs and prognostically significant genes was identified as hub genes for further analysis. For our hub genes, Kaplan-Meier (KM) survival curves were plotted to demonstrate their prognostic effects. The analysis was conducted using the survival package to perform the proportional hazards assumption test and fit the Cox proportional hazards regression models. The results were visualized using the survminer and ggplot2 packages. When employing the optimal grouping method, the surv_cutpoint function from the survminer package was utilized to determine the best cut-off points. The prognostic type assessed was Overall Survival (OS). To analyze the correlations among hub genes, we utilized the circlize package (version 0.4.1) in R. The process involved calculating the pairwise correlations between variables in our dataset and visualizing the results using the circlize package. The statistical method employed for this analysis was Spearman’s rank correlation. Additionally, we performed separate correlation analyses for different OS events (alive or dead) to compare the correlation patterns in these two groups. This methodological approach allowed us to comprehensively assess and visualize the relationships among hub genes under varying survival outcomes. To evaluate the diagnostic efficacy of hub genes, we utilized the pROC package (version 1.18.0) in R. The process involved performing Receiver Operating Characteristic (ROC) analysis using the pROC package, followed by plotting the ROC curves to visualize the diagnostic performance of each gene.

### Human prostate samples

Human prostate samples from anonymous PCa patients and normal controls were obtained from Department of Urology, Shuguang Hospital Affiliated to Shanghai University of Traditional Chinese Medicine. Informed consent was obtained from all subjects. The study was approved by the ethics review board of Shuguang Hospital (Approval Number: 2022-1210-147-02). The study abided by the Declaration of Helsinki principles.

### Gene expression analysis

Gene expression analysis was conducted using clinical tissues obtained from PCa and normal tissues. Quantitative polymerase chain reaction (qPCR) was performed to validate the expression of protein-coding hub genes. The experimental procedure began with the extraction of total RNA from the tissues using the TRIzol reagent, followed by the purification of RNA with a RNeasy Mini Kit. The concentration and purity of RNA were measured using a NanoDrop spectrophotometer. Subsequently, 1 µg of RNA was reverse-transcribed into complementary DNA (cDNA) using a High-Capacity cDNA Reverse Transcription Kit. qPCR was carried out using SYBR Green Master Mix on an ABI 7500 Real-Time PCR System. Specific primers for the hub genes were designed, and the relative expression levels were calculated using the 2^^-ΔΔCt^ method, with GAPDH as the internal control.

### Immunohistochemical analysis

Tissue sections were deparaffinized in xylene, rehydrated through a graded ethanol series, and subjected to antigen retrieval in a citrate buffer. Endogenous peroxidase activity was blocked with hydrogen peroxide, and the sections were incubated with primary antibodies specific to the hub genes at 4°C overnight. After washing, the sections were incubated with biotinylated secondary antibodies, followed by streptavidin-horseradish peroxidase conjugate. The chromogenic detection was performed using a DAB substrate kit, and the sections were counterstained with hematoxylin, dehydrated, and mounted. The stained sections were examined under a light microscope, and the expression levels of the hub genes were assessed based on the intensity and extent of staining.

### Cell culture and treatments

PC-3(CVCL_0035, purchased from Wuhan Pricella Biotechnology Co., Ltd.) and DU145(CVCL_0105, purchased from Baidi Biotech Ltd.) cells were maintained in RPMI-1640 medium supplemented with 10% fetal bovine serum (FBS, v/v), 100 U/mL penicillin, and 100 μg/mL streptomycin under standard culture conditions (37°C, 5% CO_2_, humidified atmosphere). All cell lines were authenticated using short tandem repeat (STR) profiling within the last 3 years. All experiments were performed with mycoplasma‐free cells. BAIAP2L2 expression was suppressed using short hairpin RNA (shRNA-BAIAP2L2), with their negative control (sh-NC) serving as the experimental control. Lentiviral vectors carrying BAIAP2L2-targeted shRNA were constructed according to the manufacturer’s specifications and subsequently used to infect PC3 and DU145 cells. Stable cell lines exhibiting reduced BAIAP2L2 expression were validated through RT-qPCR analyses prior to subsequent experimental procedures.

### Cell proliferation assay

Cell proliferation was quantitatively assessed using the Cell Counting Kit-8 (CCK-8) assay. Cells were seeded in 96-well plates at a density of 3,000 cells per well in triplicate and allowed to adhere for 24, 48 and 72 hours, followed by the addition of 10 µl CCK-8 reagent to each well. Following a 2-hour incubation period under dark conditions at 37°C, absorbance measurements were recorded at 450 nm wavelength using a microplate spectrophotometer.

Clonogenic potential was evaluated through colony formation assays. Logarithmic-phase cells from each experimental group were enzymatically dissociated using trypsin and seeded at a density of 500 cells per 35 mm culture dish, followed by continuous culture for 2–3 weeks. Upon visible colony formation, cultures were terminated and fixed with 4% paraformaldehyde for 20 minutes, followed by crystal violet staining for 30 minutes. Colony quantification was performed microscopically, and the colony formation rate was calculated as the ratio of colony number to initially seeded cells, expressed as a percentage.

### Cell migration assay

Cell migration assay was evaluated using the scratch assay. Stably transfected PC3 and DU145 cells (shRNA-BAIAP2L2 and sh-NC groups) were seeded in 6-well plates at a density of 1 × 10^6^ cells per well and cultured for 24 hours to achieve confluence. Uniform scratches were created using a 200 μL pipette tip, followed by three successive PBS washes to remove dislodged cells. After 24 hours of incubation in fresh medium, images of cell migration into the wounded area were captured at identical positions to assess wound closure rates.

### Pathway analysis

To further investigate the molecular mechanisms underlying function in PCa of key genes, we conducted pathway enrichment and Gene Ontology (GO) analysis using GeneCards (www.genecards.org), including the Reactome pathway analysis, GO enrichment analysis and protein-protein interactions (PPIs) using the STRING database (www.string-db.org).

### Statistical analyses

Statistical analyses were performed using Statview software (SAS Institute, Calabasas, CA). Data are presented as mean ± SEM. Statistical comparisons between groups were conducted using two-tailed independent Student’s t-tests or one-way analysis of variance (ANOVA) followed by Fisher’s *post hoc* tests where appropriate. Statistical significance was established at p < 0.05.

## Results

### Transcriptomic data analysis of DEGs

**Figure 1:**
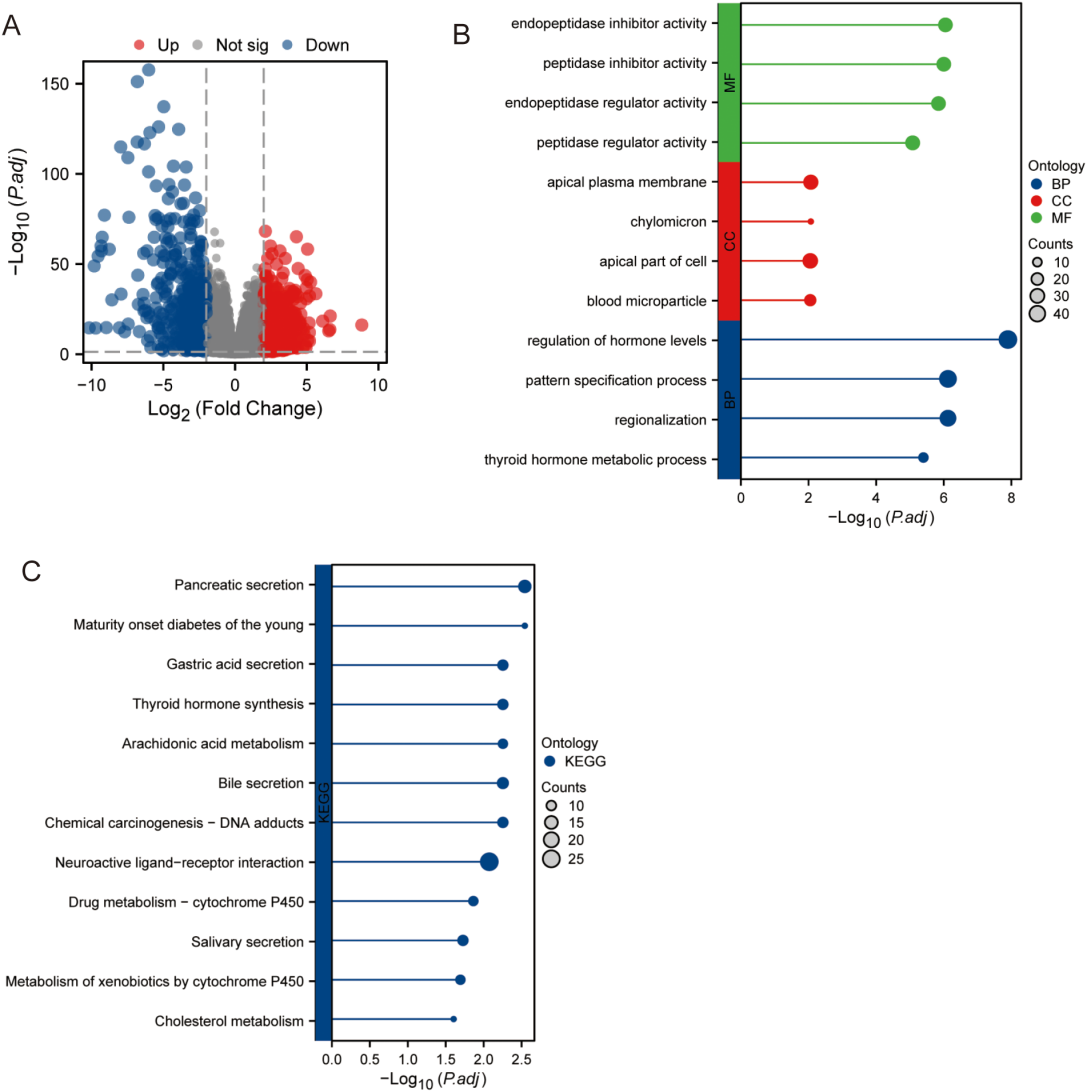
Gene expression in PCa samples and normal tissue samples. **(A)** Expression of different genes. **(B)** GO analysis. **(C)** KEGG analysis.

As shown in **Figure 1A**, 775 genes were upregulated, and 674 genes were downregulated. Prognostic analysis revealed that 748 genes were associated with clinical outcomes. As shown in **Figure 1B**, the analysis of our differentially expressed genes revealed significant associations with several key biological pathways. These genes were primarily involved in the regulation of hormone levels, thyroid hormone metabolic processes, and various cellular processes such as the apical plasma membrane and cell parts. Additionally, significant associations were observed with enzyme regulatory activities, particularly peptidase and endopeptidase inhibitor activities. On the pathway level, our data highlighted critical metabolic and secretion pathways, including cholesterol metabolism, metabolism of xenobiotics by cytochrome P450, and bile and pancreatic secretions. Notably, pathways related to drug metabolism, thyroid hormone synthesis, and maturity-onset diabetes of the young were also enriched (**Figure 1C**). This comprehensive pathway enrichment underscores the multifaceted roles of our differentially expressed genes in metabolic and regulatory networks.

### The GSEA of differentially expressed genes

**Figure 2:**
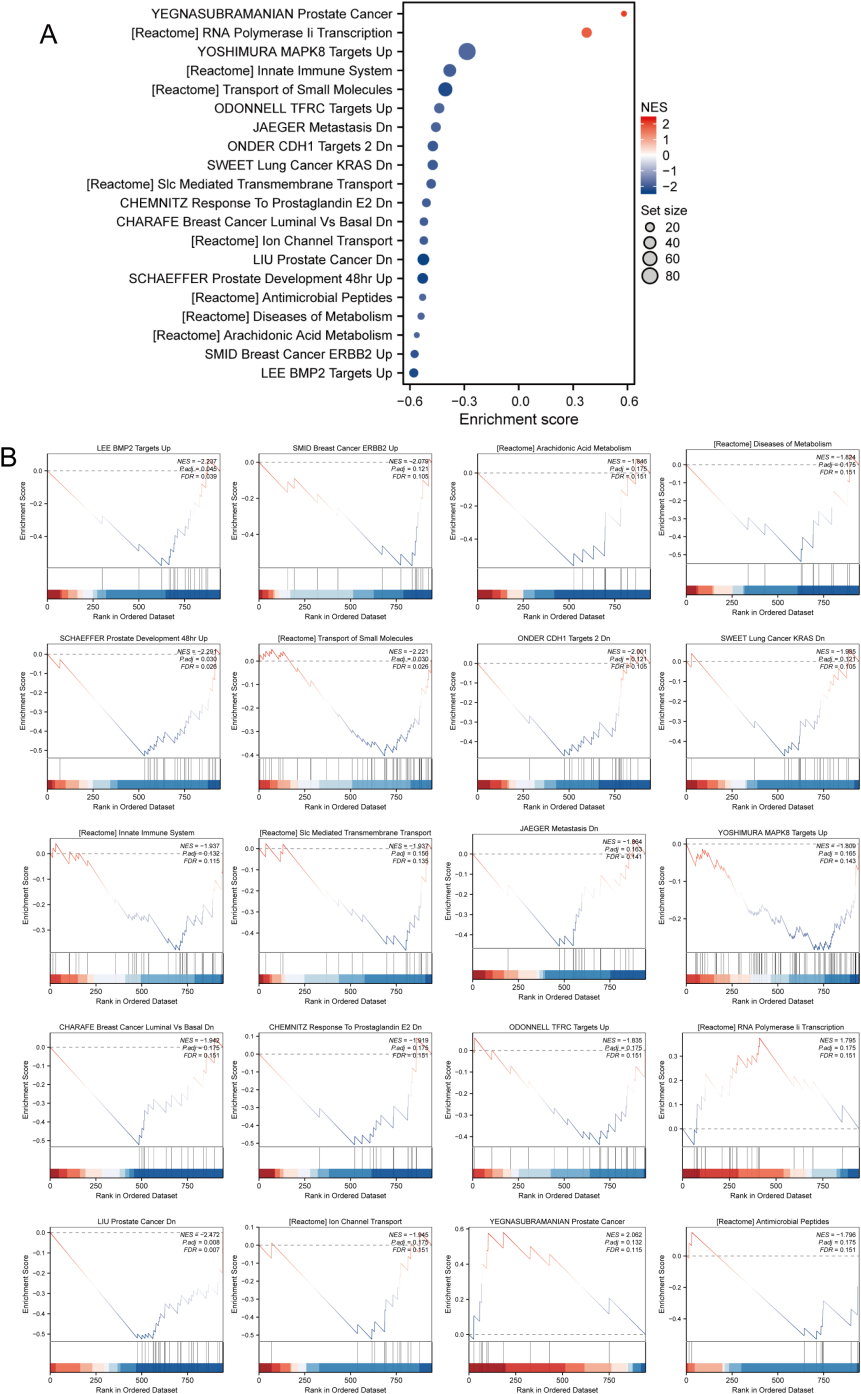
The result of GSEA in differentially expressed genes. **(A)** Key pathways and gene sets. **(B)** Detailed information on gene expression.

As shown in **Figure 2A**, the GSEA of our differentially expressed genes revealed several significant pathways and gene sets. Notably, pathways such as BMP2 targets, ERBB2 in breast cancer, and arachidonic acid metabolism were enriched, indicating their upregulation. Furthermore, metabolic diseases, antimicrobial peptides, and ion channel transport pathways from the Reactome database were significantly associated. Specific cancer-related gene sets, including PCa down-regulation, lung cancer with KRAS down-regulation, and breast cancer luminal versus basal down-regulation, were highlighted. Additional significant associations included innate immune system pathways, RNA polymerase II transcription, and the transport of small molecules (**Figure 2B**). This GSEA underscores the diverse biological processes and pathways involved, reflecting the complexity of the underlying genetic mechanisms.

### The intersection of the prognostically significant genes and DEGs

**Figure 3:**
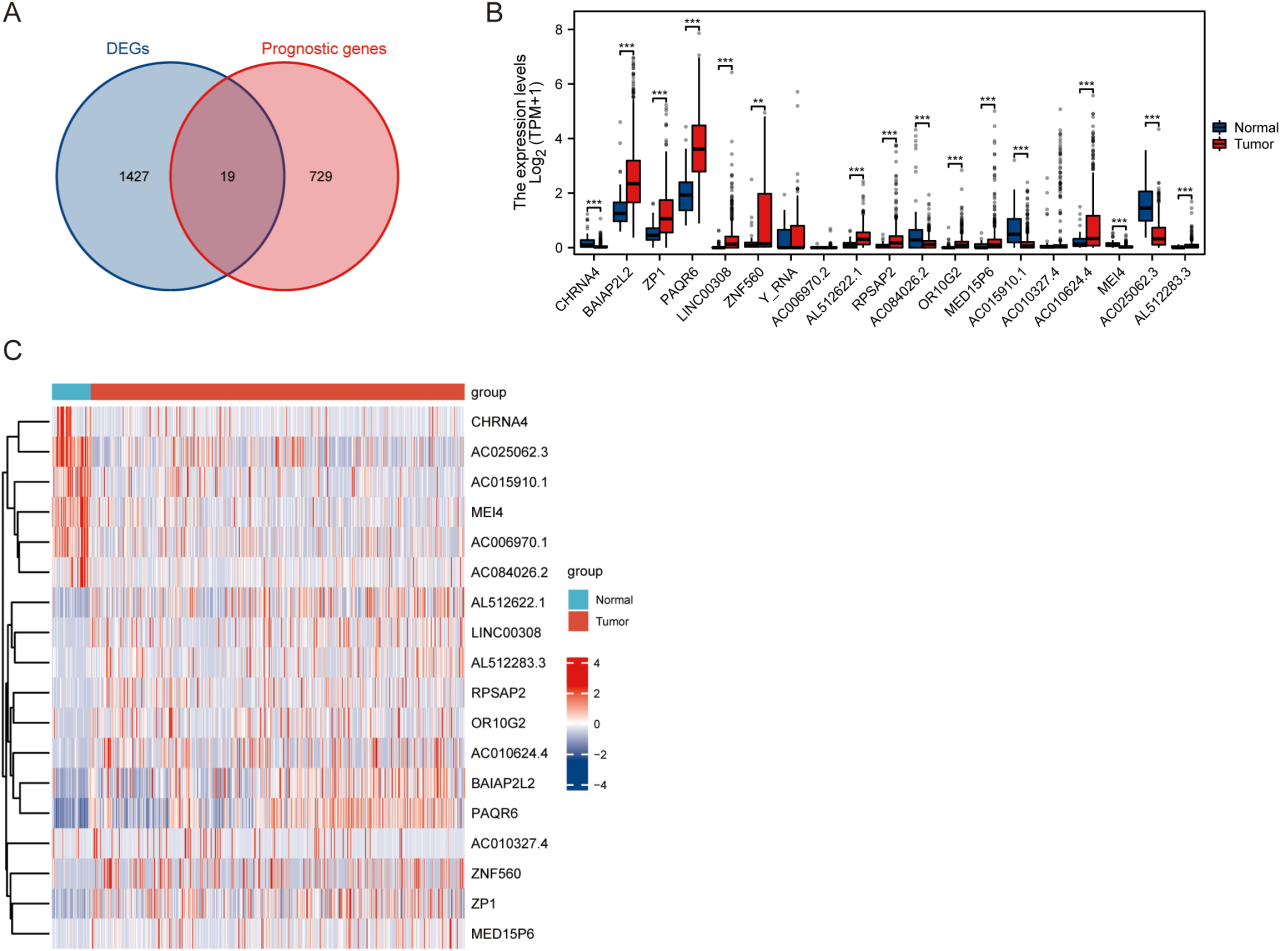
Differential expression analysis of hub genes in normal and tumor samples. **(A)** Intersection of prognostic genes and DEGs. **(B)** Statistical analysis of differential expression between normal and tumor samples of hub genes. **(C)** Heatmap displaying the relative expression levels of the hub genes across normal and tumor samples. ** P<0.01, *** P<0.001.

As depicted in **Figures 3A**, the intersection of the prognostically significant genes and DEGs identified 19 hub genes (**Supplementary Table S1**). Analysis of the 19 hub genes, as illustrated in **Figures 3B, C**, revealed statistically significant differences in expression between tumor and normal tissue samples. Specifically, CHRNA4, BAIAP2L2, ZP1, PAQR6, LINC00308, ZNF560, AL512622.1, RPSAP2, AC084026.2, OR10G2, MED15P6, AC015910.1, AC010624.4, MEI4, AC025062.3, and AL512283.3 demonstrated statistically significant changes in expression levels. Furthermore, a distinct pattern of differential expression was observed between tumor and normal samples. Five genes - CHRNA4, AC084026.2, AC015910.1, MEI4, and AC025062.3 - exhibited decreased expression levels in tumor samples compared to normal tissue. Conversely, the remaining hub genes showed elevated expression levels in tumor samples relative to normal tissue. These findings highlight the potential functional relevance of these hub genes in tumor biology. The downregulation of CHRNA4, AC084026.2, AC015910.1, MEI4, and AC025062.3 in tumor samples suggests that these genes may act as tumor suppressors, while the upregulation of the other identified hub genes implies a possible oncogenic role.

### Overall survival of hub gene

**Figure 4:**
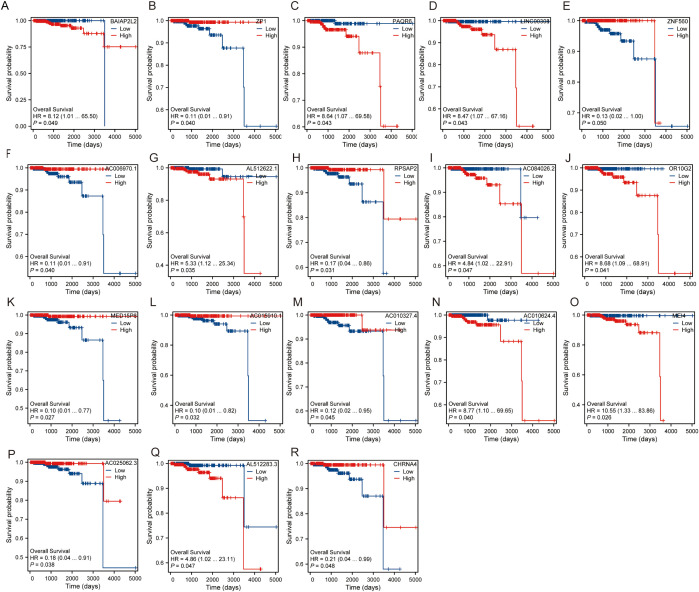
Overall survival of hub gene. **(A)** Survival probability of BAIAP2L2. **(B)** Survival probability of ZP1. **(C)** Survival probability of PAQR6. **(D)** Survival probability of LINC00308. **(E)** Survival probability of ZNF560. **(F)** Survival probability of AC006970.1. **(G)** Survival probability of AL512622.1. **(H)** Survival probability of RPSAP2. **(I)** Survival probability of AC084026.2. **(J)** Survival probability of OR10G2. **(K)** Survival probability of MED15P6. **(L)** Survival probability of AC015910.1. **(M)** Survival probability of AC010327.4. **(N)** Survival probability of AC010624.4. **(O)** Survival probability of MEI4. **(P)** Survival probability of AC025062.3. **(Q)** Survival probability of AL512283.3. **(R)** Survival probability of CHRNA4.

For each hub gene, we assessed Overall Survival (OS) using Kaplan-Meier survival curves and Cox proportional hazards regression analysis. The hazard ratios (HR) and p-values for each gene are as follows: ZNF560 (HR = 0.13, 95% CI: 0.02-1.00, P = 0.050), OR10G2 (HR = 8.68, 95% CI: 1.09-68.91, P = 0.041), AL512622.1 (HR = 5.33, 95% CI: 1.12-25.34, P = 0.035), AC006970.1 (HR = 0.11, 95% CI: 0.01-0.91, P = 0.040), BAIAP2L2 (HR = 8.12, 95% CI: 1.01-65.50, P = 0.049), RPSAP2 (HR = 0.17, 95% CI: 0.04-0.86, P = 0.031), ZP1 (HR = 0.11, 95% CI: 0.01-0.91, P = 0.040), LINC00308 (HR = 8.47, 95% CI: 1.07-67.16, P = 0.043), PAQR6 (HR = 8.64, 95% CI: 1.07-69.58, P = 0.043), AC084026.2 (HR = 4.84, 95% CI: 1.02-22.91, P = 0.047), AC015910.1 (HR = 0.10, 95% CI: 0.01-0.82, P = 0.032), MEI4 (HR = 10.55, 95% CI: 1.33-83.86, P = 0.026), AL512283.3 (HR = 4.86, 95% CI: 1.02-23.11, P = 0.047), AC010624.4 (HR = 8.77, 95% CI: 1.10-69.65, P = 0.040), CHRNA4 (HR = 0.21, 95% CI: 0.04-0.99, P = 0.048), AC010327.4 (HR = 0.12, 95% CI: 0.02-0.95, P = 0.045), MED15P6 (HR = 0.10, 95% CI: 0.01-0.77, P = 0.027), AC025062.3 (HR = 0.18, 95% CI: 0.04-0.91, P = 0.038) (**Figures 4A–R**). Among these, genes such as ZNF560, AC006970.1, RPSAP2, ZP1, AC015910.1, CHRNA4, AC010327.4, MED15P6, and AC025062.3 exhibited a protective effect with hazard ratios significantly less than 1, indicating that higher expression of these genes is associated with improved overall survival. Conversely, genes including OR10G2, AL512622.1, BAIAP2L2, LINC00308, PAQR6, MEI4, AL512283.3, and AC010624.4 had hazard ratios greater than 1, suggesting that higher expression levels of these genes are associated with poorer overall survival. These findings highlight the diverse roles these hub genes play in influencing patient outcomes and underscore their potential as prognostic biomarkers.

### The correlation analysis of hub genes

**Figure 5:**
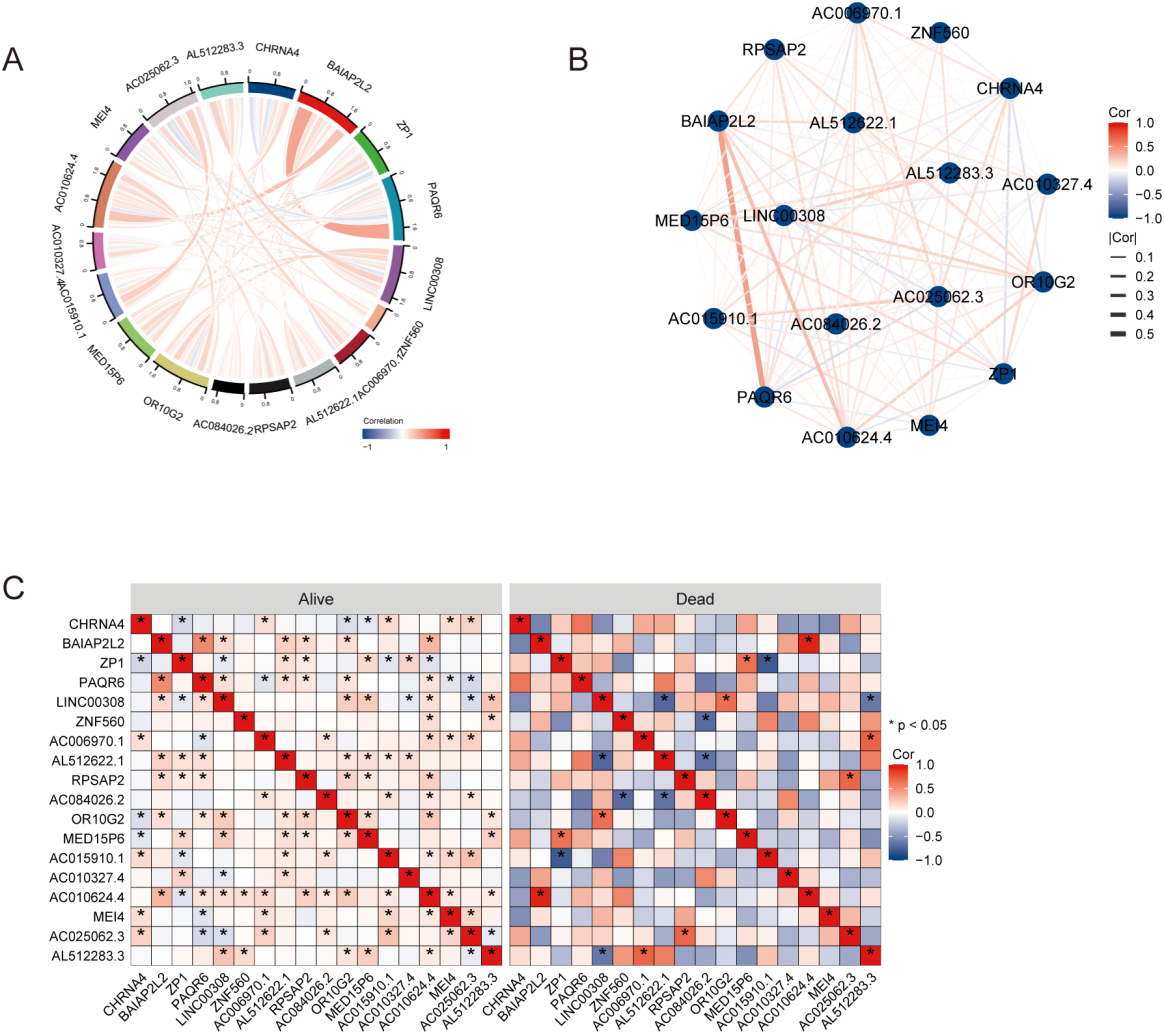
The correlation analysis of hub genes. **(A)** Chord diagrams. **(B)** Network graphs. **(C)** Heatmaps. * P<0.05.

The correlation analysis among hub genes was performed and were visualized using chord diagrams, network graphs and heatmaps (**Figures 5A–C**). Spearman’s rank correlation was employed to determine the pairwise correlations between the hub genes. The chord diagram revealed notable interactions among genes such as CHRNA4, BAIAP2L2, ZP1, and PAQR6, displaying both positive and negative correlations (**Figure 5A**). The heatmap provided a detailed view of these correlations, highlighting significant relationships with varying degrees of strength. Additionally, the analysis was conducted separately for different OS events (alive or dead). The heatmap comparison between the two groups illustrated distinct correlation patterns. For example, some genes exhibited stronger correlations in the “alive” group compared to the “dead” group, suggesting potential differences in gene interactions depending on the survival status of patients (**Figure 5C**). These results indicate that the hub genes are part of complex interaction networks that influence overall survival. The differential correlation patterns between the alive and dead groups suggest that specific gene interactions may play crucial roles in determining patient outcomes, highlighting their potential as prognostic biomarkers.

### The diagnostic performance of each hub gene

**Figure 6:**
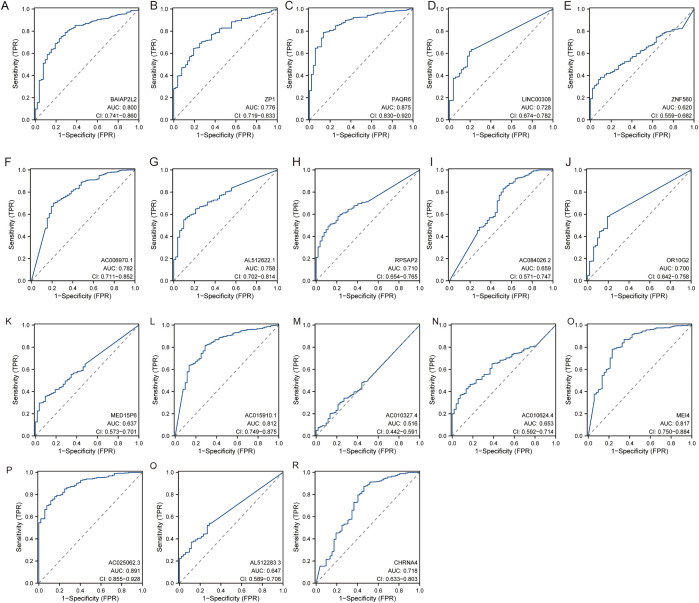
The diagnostic performance of each hub gene. **(A)** BAIAP2L2. **(B)** ZP1. **(C)** PAQR6. **(D)** LINC00308. **(E)** ZNF560. **(F)** AC006970.1. **(G)** AL512622.1. **(H)** RPSAP2. **(I)** AC084026.2. **(J)** OR10G2. **(K)** MED15P6. **(L)** AC015910.1. **(M)** AC010327.4. **(N)** AC010624.4. **(O)** MEI4. **(P)** AC025062.3. **(Q)** AL512283.3. **(R)** CHRNA4.

The diagnostic performance of each hub gene was evaluated using Receiver Operating Characteristic analysis, with the following results: ZP1 had an AUC of 0.776 (CI: 0.719−0.833), PAQR6 showed an AUC of 0.875 (CI: 0.830−0.920), ZNF560 had an AUC of 0.620 (CI: 0.559−0.682), LINC00308 showed an AUC of 0.728 (CI: 0.674−0.782), RPSAP2 had an AUC of 0.710 (CI: 0.654−0.765), OR10G2 displayed an AUC of 0.700 (CI: 0.642−0.758), AC006970.1 had an AUC of 0.782 (CI: 0.711−0.852), AL512622.1 showed an AUC of 0.758 (CI: 0.702−0.814), AC084026.2 had an AUC of 0.659 (CI: 0.571−0.747), BAIAP2L2 displayed an AUC of 0.800 (CI: 0.741−0.860), MEI4 had an AUC of 0.817 (CI: 0.750−0.884), CHRNA4 showed an AUC of 0.718 (CI: 0.633−0.803), MED15P6 had an AUC of 0.637 (CI: 0.573−0.701), AC015910.1 showed an AUC of 0.812 (CI: 0.749−0.875), AC010327.4 had an AUC of 0.516 (CI: 0.442−0.591), AC010624.4 displayed an AUC of 0.653 (CI: 0.592−0.714), AC025062.3 had an AUC of 0.891 (CI: 0.855−0.928), and AL512283.3 showed an AUC of 0.647 (CI: 0.589−0.706) (**Figure 6**). These results indicate varying degrees of diagnostic accuracy, with genes like PAQR6, AC025062.3, and MEI4 showing particularly high diagnostic performance, as reflected by their higher AUC values.

### Validating the hub gene

**Figure 7:**
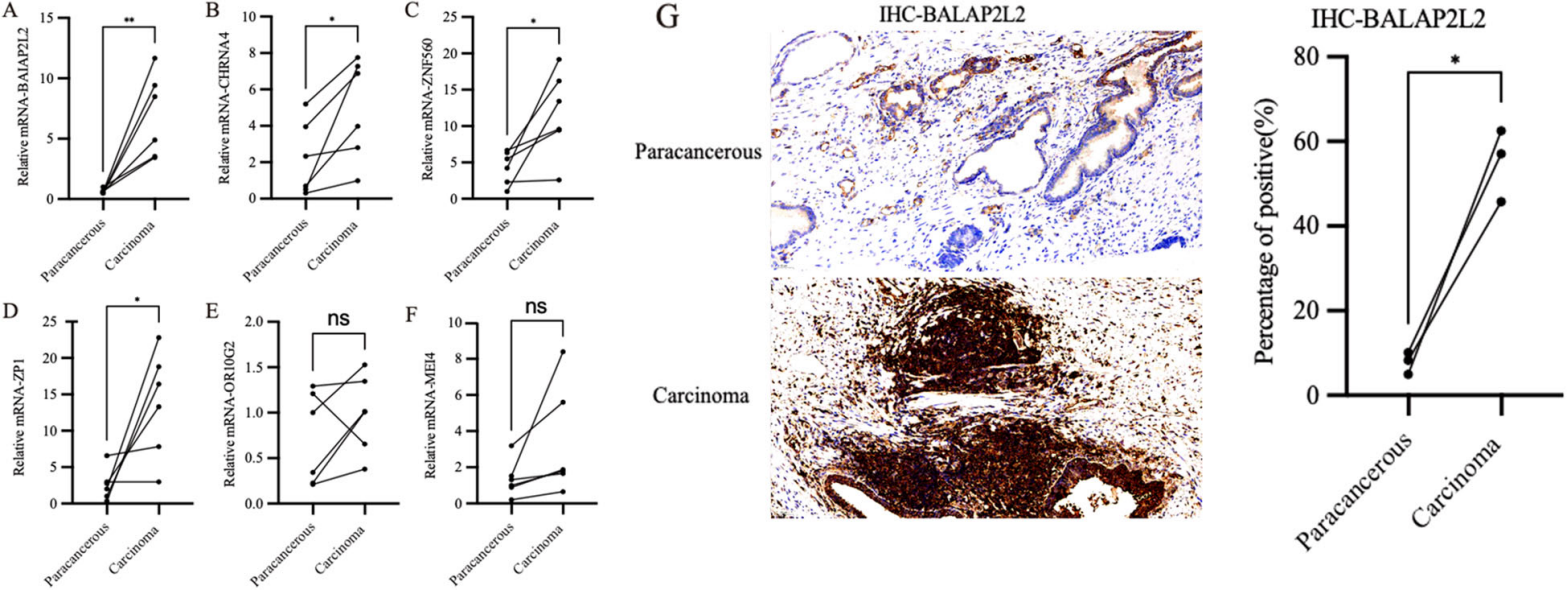
Expression profiles of hub genes in clinical samples. **(A)** The expression levels of BAIAP2L2 genes. **(B)** The expression levels of CHRNA4 genes. **(C)** The expression levels of ZNF560 genes. **(D)** The expression levels of ZP1 genes. **(E)** The expression levels of OR10G2 genes. **(F)** The expression levels of MEI4 genes. **(G)** IHC analysis of BAIAP2L2. * P<0.05, ** P<0.01, ns P>0.05.

Based on the identified hub genes, we conducted qPCR analysis to compare the expression levels of CHRNA4, BAIAP2L2, ZP1, OR10G2, ZNF560 and MEI4 between clinical PCa samples and normal tissues. As shown in **Figures 7A–F**, the expression levels of four genes—CHRNA4, BAIAP2L2, ZP1, and ZNF560—were significantly higher in PCa tissues compared to normal tissues, corroborating the findings from transcriptomic data analysis. Immunohistochemistry further validated these results, as shown in **Figure 7G**. The IHC analysis indicated that the proteins encoded by three of these genes, including BAIAP2L2, were expressed at higher levels in PCa tissues relative to normal tissues. These findings highlight the potential involvement of these genes in the pathogenesis of PCa and suggest their utility as biomarkers for the disease.

### Effects of BAIAP2L2 on PC3 and DU145 cell function

**Figure 8:**
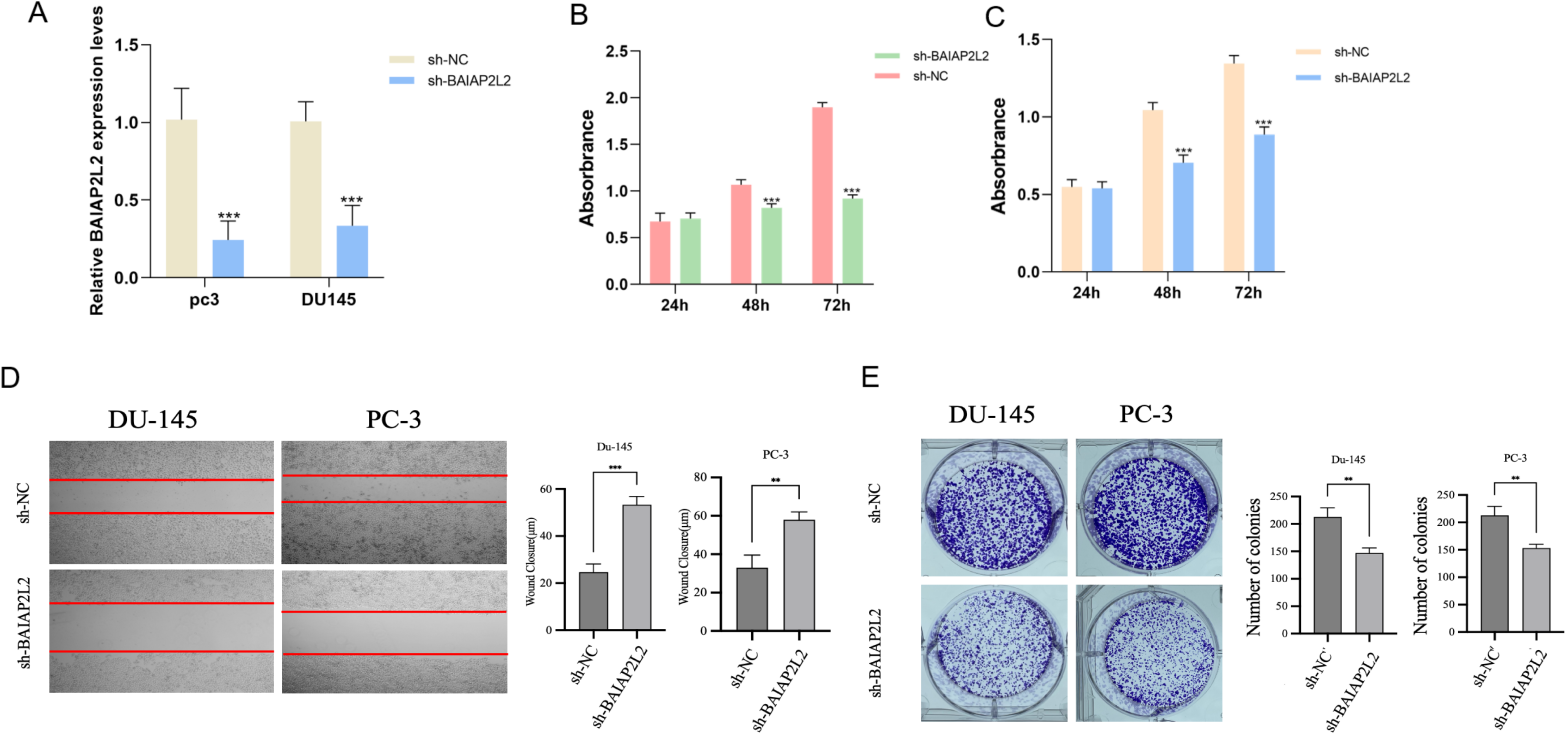
Effect of BAIAP2L2 on the function of PC3 and DU145 cells. **(A)** Relative mRNA expression level of BAIAP2L2 in DU145 and PC3 cells. **(B, C)** Comparison of cell proliferation between sh- BAIAP2L2 group and Sh-NC group in DU145 and PC3 cells. **(D)** The wound healing assay. **(E)** The colony formation assay. ** P<0.01, *** P<0.001.

The knockdown efficiency of BAIAP2L2 in PC3 and DU145 cells was validated through both qPCR and Western blot analyses (**Figures 8A**). Cell proliferation assays using CCK-8 demonstrated that BAIAP2L2-depleted cells exhibited significantly reduced proliferation rates compared to the sh-NC groups in both PC3 and DU145 cell lines (**Figures 8B, C**). The wound healing assay results, depicted in **Figure 8D**, demonstrated a significant reduction in the migration capability of BAIAP2L2 knockdown cells, as evidenced by slower wound closure rates over the observed time points. The colony formation assay, as illustrated in **Figure 8E**, showed that the number of colonies formed by BAIAP2L2 knockdown cells was significantly lower than that of the sh-NC groups, indicating impaired cell proliferation. Taken together, these experimental observations provide compelling evidence that BAIAP2L2 serves as a crucial regulator of both proliferative and migratory phenotypes in PCa cells, highlighting its potential mechanistic involvement in disease progression.

### Pathway analysis of BAIAP2L2

Additionally, we performed a pathway and GO analysis using GeneCards (www.genecards.org). Our analysis revealed that BAIAP2L2 is involved in key cellular pathways, particularly Rho GTPase cycle, RHOF GTPase cycle, and signal transduction pathways. These pathways are critical for cytoskeletal organization, cell motility, and intracellular signaling, which align with our experimental findings on BAIAP2L2-mediated proliferation and migration in PCa cells. Furthermore, the Gene Ontology (GO) analysis identified BAIAP2L2 as being involved in plasma membrane organization (GO:0007009), actin filament polymerization (GO:0030838), and actin filament bundle assembly (GO:0051017). These biological processes are essential for cell shape maintenance, adhesion, and motility, further supporting the hypothesis that BAIAP2L2 enhances the metastatic potential of PCa cells via actin cytoskeletal remodeling. Additionally, the STRING protein interaction analysis highlighted several interacting partners, including ALG3, ALG5, ALG12, DPM1, and RPN1, which are involved in protein glycosylation and intracellular transport. This suggests a potential link between BAIAP2L2 activity and glycoprotein biosynthesis, which warrants further exploration in future studies.

## Discussion

In our study, transcriptome data analysis revealed a substantial number of differentially expressed genes in PCa tissues compared to normal tissues, with 775 genes upregulated and 674 genes downregulated. The prognostic analysis further pinpointed 748 genes associated with clinical outcomes, and the intersection of DEGs and prognostically significant genes highlighted 19 hub genes. BAIAP2L2, an IRSp53 family protein containing the characteristic BAR domain, was found to localize to Rab13-positive vesicles and plasma membrane junctions (20). Unlike typical BAR domains, it does not induce membrane deformation but instead facilitates the formation of planar membrane sheets (21). Recent studies have increasingly implicated BAIAP2L2 in the progression of various malignancies. BAIAP2L2 promotes gastric cancer cell proliferation and metastasis via activation of AKT/mTOR and Wnt3α/β-catenin pathways, while serving as a prognostic marker in non-small cell lung cancer patients with low PD-1 and EGFR expression (22, 23). In PCa cells, BAIAP2L2 may influence tumor initiation and malignant progression through modulation of VEGF signaling and apoptotic pathways (24). The human oocyte is enveloped by the zona pellucida (ZP), a translucent fibrillar network composed of four glycoproteins (ZP1-4), with ZP1 serving as a crucial crosslinker that maintains ZP structural integrity (25). Studies have demonstrated that ZP1 is expressed in PCa cell lines such as PC3, while the ZP family has been shown to significantly influence tumor cell viability, proliferation, and migration rates (26, 27). Zinc finger protein 560 (ZNF560), a member of the zinc finger protein family, plays crucial roles in various biological processes, including development, differentiation, metabolism, and apoptosis, and is closely associated with different stages of cancer progression (28). OR10G2, a member of the olfactory receptor family, belongs to the G protein-coupled receptor (GPCR) superfamily encoded by single-exon genes (29). GPCRs play crucial roles in diverse physiological functions. Activated by various molecules including hormones, lipids, peptides, and neurotransmitters, GPCRs couple with specialized transducer proteins known as G proteins, initiating multiple signaling pathways (30). These coordinated signaling pathways trigger biochemical reactions that influence multiple pathophysiological processes, including cancer development. MEI4 is crucial for the formation of DNA double-strand breaks (DSBs) and localizes as foci on chromosome axes at the onset of meiotic prophase (31). Aberrant expression of meiotic genes in cancer cells has been shown to promote various hallmarks of cancer by altering centromere polarity control, motility, chromosome dynamics, and DNA repair mechanisms (32). CHRNA4 encodes a nicotinic acetylcholine receptor (nAChR), a member of the ligand-gated ion channel superfamily that mediates rapid synaptic signal transmission (33). By integrating transcriptomic data from TCGA with differential expression and prognostic analyses, we identified key genes associated with PCa progression. This finding underscores the complexity of the molecular mechanisms driving PCa and the pivotal role these hub genes might play in tumorigenesis.

Subsequently, in combination with clinical samples, CHRNA4, BAIAP2L2, ZP1 and ZNF560 were expressed at significantly higher levels in PCa tissues compared to normal tissues, confirming the relevance of these genes to PCa. Immunohistochemical analysis of BAIAP2L2 expression revealed significantly elevated levels of BAIAP2L2, in PCa tissues. Previous studies have demonstrated that BAIAP2L2 is overexpressed in gastric cancer and associated with metastasis, whereby knockdown of BAIAP2L2 suppresses the activation of the Wnt signaling pathway, consequently inhibiting gastric cancer cell proliferation and metastasis (23). Consequently, functional assays performed on PC3 and DU145 cell lines, including wound healing, colony formation, and CCK-8 proliferation assays, demonstrated that knockdown of BAIAP2L2 significantly impaired cell migration, proliferation, and viability. Functional assays such as the wound healing assay demonstrated that BAIAP2L2 knockdown significantly impairs cell migration, as evidenced by slower wound closure rates. This reduction in migratory capability suggests that BAIAP2L2 is essential for the metastatic potential of PCa cells. The colony formation assay showed a marked decrease in the number of colonies formed by BAIAP2L2 knockdown cells, indicating reduced cell proliferation. This was corroborated by the CCK-8 assay, which revealed a significant decrease in cell viability in BAIAP2L2 knockdown cells compared to controls. These results collectively highlight the crucial role of BAIAP2L2 in promoting cell proliferation and survival in PCa.

Furthermore, considering the significant role of BAIAP2L2 in promoting PCa cell proliferation, migration, and survival, it presents itself as a potential therapeutic target. Given its involvement in multiple oncogenic pathways, including VEGF signaling and Wnt/β-catenin pathways, targeting BAIAP2L2 could disrupt critical mechanisms underlying PCa progression. Small molecule inhibitors, monoclonal antibodies, or RNA-based therapies such as siRNA or antisense oligonucleotides could be explored to suppress BAIAP2L2 expression or activity. Previous studies have demonstrated the efficacy of targeting the Wnt/β-catenin pathway in various cancers (34), suggesting that pharmacological modulation of BAIAP2L2-associated signaling might enhance the efficacy of existing PCa therapies. Additionally, its differential expression in tumor versus normal tissues highlights its potential utility as a biomarker for early diagnosis and prognosis. Future studies should focus on preclinical and clinical investigations to assess the therapeutic feasibility of BAIAP2L2 inhibition, its synergy with existing therapies (e.g., androgen deprivation therapy and immune checkpoint inhibitors), and its impact on tumor microenvironment interactions. Such efforts would pave the way for translating these findings into clinical applications, potentially leading to novel treatment strategies for PCa patients. In addition to its role in promoting PCa progression, BAIAP2L2 also holds promise as a prognostic and diagnostic biomarker. Compared to traditional PCa biomarkers such as prostate-specific antigen (PSA) and androgen receptor (AR) variants, BAIAP2L2 may offer complementary value, particularly in cases where PSA levels lack specificity or AR variants contribute to therapy resistance. While PSA remains the gold standard for PCa screening, its low specificity often leads to unnecessary biopsies. Emerging evidence suggests that gene expression-based biomarkers, like BAIAP2L2, could improve risk stratification and disease monitoring. Given its significant correlation with tumor progression and patient survival, BAIAP2L2 could potentially enhance patient stratification for aggressive versus indolent PCa, aiding in personalized treatment decisions. Future studies should evaluate BAIAP2L2’s predictive accuracy in large patient cohorts and explore whether it can serve as an independent predictor or work in conjunction with existing biomarkers to refine PCa prognosis and treatment selection.

While our study provides valuable insights into the role of BAIAP2L2 and other hub genes in PCa progression, several limitations should be acknowledged. First, our transcriptomic analysis relied primarily on TCGA database, which may not fully represent the global patient population’s genetic diversity. Future studies should incorporate data from multiple cohorts across different ethnic backgrounds to validate the universal applicability of these findings. Second, although we demonstrated the functional significance of BAIAP2L2 through *in vitro* experiments, our study lacks comprehensive *in vivo* validation through animal models. The absence of xenograft or transgenic mouse experiments limits our ability to fully characterize the role of BAIAP2L2 in tumor growth, progression, and metastasis within the complex tumor microenvironment. Third, the precise molecular mechanisms by which BAIAP2L2 regulates PCa progression remain to be fully elucidated. Our current study establishes the phenotypic consequences of BAIAP2L2 knockdown but does not comprehensively map the downstream signaling pathways affected by BAIAP2L2 modulation. Future investigations should explore potential interactions with key oncogenic pathways in PCa, such as androgen receptor signaling, PI3K/AKT/mTOR pathway, or Wnt/β-catenin signaling, which could provide deeper insights into how BAIAP2L2 influences tumor cell behavior. Finally, our study focused mainly on cell lines and clinical samples at specific time points, lacking longitudinal data that could better illustrate the dynamic changes in gene expression during disease progression. These limitations highlight important directions for future research, including *in vivo* validation using appropriate animal models to confirm the role of BAIAP2L2 in PCa progression; more detailed mechanistic studies to elucidate the molecular pathways mediated by BAIAP2L2; investigation of potential interaction partners of BAIAP2L2 that might contribute to its regulatory role in PCa; and exploration of combinatorial approaches targeting multiple hub genes to maximize therapeutic impact. Such comprehensive investigations would significantly enhance our understanding of the molecular mechanisms driving PCa and could potentially lead to more effective targeted therapies.

## Data Availability

The original contributions presented in the study are included in the article/**Supplementary Material**. Further inquiries can be directed to the corresponding authors.
